# The evolutionary characteristics and neighbourhood mechanisms of urban innovation networks: The case of China’s sports industry

**DOI:** 10.1371/journal.pone.0319732

**Published:** 2025-03-31

**Authors:** Kun Peng, Yimeng Ma, Laibing Lu, Dangsheng Wang, Dong Zhu

**Affiliations:** 1 Department of Sports, Henan Institute of Technology, Xinxiang, China; 2 College of Innovation and Entrepreneurship, Xinxiang Medical University, Xinxiang, China; 3 School of Physical Education and Sport Science, Fujian Normal University, Fuzhou, China; 4 Department of Sports Rehabilitation, Hunan University of Medicine, Huaihua, China; Tsinghua University, CHINA

## Abstract

Taking the patents granted from 2006 to 2023 and the applicants located in different cities in China as data samples, with the help of UCINET 6.0 software and according to the timeline, we visualise the cooperation and innovation network of the sports industry during the period of the “11th Five-Year Plan” to the “14th Five-Year Plan”. QAP multiple regression analysis was used to explore the impact of geographical, cognitive, institutional, economic, and technological proximity on the collaborative innovation performance of the sports industry at different stages.The research results indicate that (1) The scale of China’s sports industry cooperation and innovation network continues to expand, the cohesion of the network is constantly strengthening, and the complexity of regional cooperation relationships is gradually increasing, forming a grid-based cooperation trend with multiple innovative entities from the early “one place leading” model; (2) From 2006 to 2015, innovation cooperation in the sports industry was more likely to occur between geographically adjacent regions. Since 2016, innovation cooperation in the sports industry has gradually broken geographical limitations. In addition, since 2011, the level of knowledge sharing among sports technology innovation entities in various cities in China has further deepened, the gap in cognitive ability has intensified the efficiency of cooperation between different innovation entities, and innovation cooperation is concentrated between cities with comparable economic strength. Starting from the “14th Five Year Plan” period, cooperation between cities with similar innovative technological capabilities has become closer, and the efficiency of innovation output has significantly improved

## 1. Introduction

The report of the twentieth CPC National Congress emphasised that science and technology innovation is an important driving force for the high-quality development of China’s economy. According to the report, the first productive force is to adhere to science and technology, talent is the first resource, the first power is innovation, and China must continue to promote scientific and technological innovation to empower high-quality development. Since the State Council of China issued the *Opinions on Accelerating the Development of the Sports Industry and Promoting Sports Consumption* [1] State Council on accelerating the development of the sports industry to promote sports consumption of a number of see.https://www.gov.cn/zhengce/zhengceku/2014-10/20/content_9152.htm,2014.]] in 2014, from 2015 to 2022, the scale of China’s sports industry has increased from 1.71 trillion yuan to 3.30 trillion yuan or 93%; the added value of the sports industry has increased from 549.4 billion yuan to 1309.2 billion yuan or as high as 138.2% [2]. The *National Fitness Programme (2021-2025)* proposes that by 2025, the total scale of the national sports industry will reach 5 trillion yuan and should strive to develop the sports industry into a pillar industry of the national economy by 2035 [3]. In the new development stage, high-quality development of the sports industry must rely on innovation-driven connotative growth [4], and scientific and technological innovation is the main engine for the sporting industry to achieve transformation and upgrading [5]. The *Programme for the Construction of a Strong Sporting Nation* also stresses “support for the research and development, design, manufacture and demonstrated application of sporting goods, and to guide enterprises to increase independent research and development and the transformation of scientific and technological achievements” [6].

However, in the global sports industry, where technological developments are becoming more complex and organisations are becoming more interconnected, the traditional “behind closed doors” approach to innovation is becoming outdated. The “flow space” formed by technology, talent, information and other resources with the help of today’s advanced network and transport has gradually deepened the degree of cooperation among innovative subjects. However, at present, the development of China’s sports industry is still in the process of gradually improving, the flow of knowledge and the efficiency of innovation among innovation players still need to be improved, and the depth and breadth of interorganisational cooperation need to be strengthened. Moreover, given the environment of accelerating technological change in the world sports industry, the internal members of the innovation network of China’s sports industry need to continuously adjust the link relationship while accelerating the research and development of technologies with independent intellectual property rights, so as to adapt to the rapidly changing internal and external competitive environment.In the new development stage of the “14th Five-Year Plan” period, it is important to determine how the innovation ability of key core technologies of China’s sports industry can be enhanced, as well as how a closely connected innovation network can be formed to promote the performance of regional cooperation and innovation in the sports industry while focusing on solving the challenges of technologies. Furthermore, it is important to understand what the theme of this development is. Around this development theme, it is necessary to first clarify the cooperation and innovation networks of China’s sports industry across different periods of time. What factors have affected urban cooperation and innovation in the sports industry? Therefore, we take microorganisations such as enterprises, colleges and universities and research institutes as the research objects, analyse the current situation of the cooperative innovation network in China’s sports industry, and examine the evolution mechanism of the cooperative innovation network of the sports industry and the influencing factors from the perspective of multidimensional proximity to provide valuable references for the high-quality development of China’s sports industry.

## 2. Literature review

### 2.1 Connotation of an innovation network and proximity mechanism

The concept of an innovation network was first proposed by Freeman, a representative of the French neighbourhood dynamics school, in 1991; this concept refers to the institutional arrangement formed by enterprises to cope with systemic innovation [7]. Subsequently, in different industrial fields around the world, the concept of an innovation network has gradually evolved into a state of connection based on innovation cooperation relationships among enterprises, universities and research institutes, which form a network structure with distinctive relationships between each other’s strengths and weaknesses, gradually developing into a stable dynamic system where resources flow to one another [8], embracing a new generation of technology-driven, demand-pulled, coupling and integration innovation [9]. Compared with the previous models, the innovation network model is relatively efficient and has a significant effect on addressing the “silo effect” of innovation activities and the transformation of local space into mobile and networked space [10].

In the process of deepening geo-economics and regional economics, scholars generally agree that the generation and evolution of regional innovation networks have complex and diverse characteristics, requiring detailed studies of collaborative innovation networks from various neighbourhood dimension perspectives. In the 1990s, the concept of “multidimensional proximity” put forwards by the French proximity dynamics school highlighted that geographical proximity is no longer the only factor influencing the flow of knowledge and innovation factors [11]. Boschma [12] classified proximity into four dimensions: geography, organisation, cognition and institution, and subsequent studies have confirmed that multidimensional proximity contributes to the evolution of innovation networks to varying degrees [13,14]. In recent years, with the in-depth study of proximity theory, economic geography and other fields have adopted the concept of “proximity” to explain the formation of industrial district networks and innovation clusters, which has further enriched the viewpoint of “multidimensional proximity” in the theory of innovation of industrial clusters and made the concept of “proximity” an increasingly important new perspective for examining cluster innovation in multidisciplinary research fields, such as regional economics, economic geography and innovation economics, at home and abroad. However, it is worth noting that proximity is not a static phenomenon, and excessive proximity may lead to the emergence of the “lock-in effect”, which in turn triggers the emergence of the “proximity paradox” phenomenon [15].

### 2.2 Research on Sports Industry Innovation Networks

China’s research on sports industry innovation started late and has mostly concerned the development strategies, influencing factors, evaluation methods, spatial patterns and other aspects of the innovation capacity and innovation performance of the sports industry. Scholars first explored the status quo and development path of the innovation capacity of the sports industry in some regions from a macro perspective [16,17],analysed the mechanism of innovation and development of China’s sports industry from different theoretical perspectives [18,19], as well as the security risks in the development of innovation [20]. However, to further enhance the innovation capacity and performance of the sports industry, it is also necessary to explore the specific influencing factors from the micro perspective, and the existing research results suggest that the internet development level [21], collaborative network capacity [22], financing mode [23], capital market support [24], blockchain technology [25], and digital economy drive [26] are all important factors. Relevant studies have further quantitatively analysed the innovation capacity and innovation performance of the sports industry in various regions [27,28]. However, owing to the imperfect statistical analyses related to the sports industry in some regions of China and the difficulty in obtaining data, the current research is mostly based on logical analyses, and in-depth empirical analyses are needed to comprehensively and accurately reflect the development trends of China’s sports industry’s innovation development.

By exploring the innovation capacity and innovation performance of the sports industry, research on innovation networks in the sports industry is not yet systematic and has not formed a clear research lineage.For example, by exploring the technology diffusion of the main network for patent research and development of sports equipment, Ming et al. [29] reported that the diffusion of patented technology within universities has improved, but the scope of dissemination is still relatively narrow; enterprises are concentrated on the profitability of their internal patent research and development, but the diffusion of the core technology is insufficient. Wang et al.[5] explored the spatial and temporal evolution characteristics of patent innovation in China’s sports industry from the perspective of geographic agglomeration and concluded that patents in the sports industry show spatial agglomeration, with high agglomeration dominating the eastern region and low agglomeration dominating the western region. In addition, Duan et al.[22] explored the relationships among the network capabilities, innovation capabilities and innovation performances of sports enterprises and reported that outwards network capability does not have a significant direct effect on the innovation performance of these enterprises. Nevertheless, it can have a significant indirect promotion effect on innovation performance through innovation capability. From the perspective of sporting events, Zhang et al.[30] reported that the social and cultural environment of people’s sports values of collaboration and trust is mainly formed and created through a series of institutional innovations by the government, sports intermediaries, event managers, colleges and other actors within the event network in a long-term joint effort.

In summary, although the importance of geographical proximity and organisational proximity has been gradually emphasised in the research on innovation networks in China’s sports industry.However, due to the incomplete statistics of China’s sports industry, it is difficult to collect,there has been a lack of rigorous quantitative research exploring their relationship with innovation performance and its mechanism of action needs to be further clarified.In addition, with the rapid evolution of technology and the expansion of global knowledge flows, the spatial structure of regional urban agglomerations has undergone profound changes. The traditional central city theory, the law of distance decay, and the hierarchical city system can no longer adequately account for the increasing demand for multilateral exchanges between cities, the expanding distance between connections, and the emerging problem of nonhierarchical spatial mobility. Over time, the repeated circulation of local knowledge can lead to its diminishing value and redundancy, thus triggering technological rigidity [31]. In this context, cross-local cooperation networks become a window for local innovation agents to access new external knowledge, market information and expertise [32]. However, the establishment and maintenance of cross-location cooperation requires a significant investment of resources due to the frictional costs associated with long-distance cooperation. Nonetheless, once an effective cross-territorial cooperation network is in place, innovators can benefit from its knowledge spillovers, update their knowledge and adjust their development strategies in a timely manner, thus reducing the risk of technological rigidity [33].In this process, cognitive proximity, institutional proximity, economic proximity and technology proximity play key roles in reducing the costs of cooperation. The discussion of these proximity factors, which has not yet been addressed in the study of China’s sports industry, is a topic worthy of in-depth exploration. Therefore, the contributions of this study are as follows:(1) Using the National Economic Industry Classification (CIC), which is more in line with China’s national conditions, we searched for patents related to China’s sports industry city cooperation from 2006 to the present, accurately quantified the level of cooperation and innovation in the sports industry in each city in China and constructed an innovative cooperation network for the periods of “11th Five-Year Plan” to the “14th Five-Year Plan”.(2) Breaking through the paradigm of previous research on the influence of geographic relationships and organisational types in the innovation network of the sports industry, we further take spatial cognition, institutional, economic and technological factors as the constituent elements of multidimensional proximity and use the quadratic assignment procedure (QAP) to explore the influences of different proximity dimensions in the process of the evolution of the innovation network of China’s sports industry with the aim of providing theoretical and practical references for the high-quality development of China’s sports industry.

## 3. Methods

This study did not involve human participants, animal experimentation, or sensitive personal data collection. All data sourced from IncoPat Technology Innovation Intelligence Platform(http://www.incopat.com), Official website of the National Bureau of Statistics(https://www.stats.gov.cn) and complied with relevant data protection regulations.

### 3.1 Data source

To study the network structure characteristics of regional innovation networks, existing literature has constructed collaborative innovation networks according to the number of joint patent applications [34], the number of thesis collaborations [35] or the number of patent transfers [36]. Among them, the number of thesis collaborations mainly focuses on the internal knowledge spillover problem, but it does not comprehensively reflect the output of scientific and technological innovation. The number of patent transfers emphasises the demand for patent applications but fails to comprehensively measure the cooperation between the subjects of collaborative innovation. The number of joint patent applications, as an important indicator for measuring the capacity of regional collaborative innovation, has become the preferred data source for constructing the collaborative innovation networks of China’s sports industry. The IncoPat database (flagship version), which contains the complete high-quality data of 158 million patent applications from more than 120 countries around the world, was adopted in this study. Some scholars have used the IPC classification numbers “A63”, “A63B” or “A43” to search for patents in the sports industry, but there are large differences between the International Economic Classification (IEC) data and the statistical classification of the sports industry in China, making it difficult to comprehensively and accurately search for China’s current sports industry patents. Therefore, this study used data from the “Sports Industry Statistical Classification (2019)” and compared it against the “National Economy Industry Classification” (GB/T 4754-2017) and the “International Patent Classification and the National Economy Industry Classification Reference Relationship Table (2018)” using the retrieval formula “CIC=(C244 OR R89 OR E4720 OR E4813 OR E4892 OR E4991)”. The start date of the analysis was set as the start year of the “11 Five-Year Plan” period, and invention patents with patent grant dates corresponding to 2006–2023 were retrieved. Moreover, according to the research purpose of this paper, patents with the applicant type “individual” without geographical and unit information, utility model patents and design patents with low technical content, and related invention patents filed by electric power systems with little relevance to sports were excluded. Finally, the screened patents were merged according to the application number to obtain 1079 patents in the sports industry with the country of disclosure as “China”, which is the basic data of this paper, among which 334 patents were located in different cities.

### 3.2 Models

This study uses social network analysis and QAP multiple regression analysis methods to study the evolution of cooperative innovation networks in the sports industry. The social network analysis method studies the interrelationships between individuals to demonstrate the position of different individuals in a network and the overall structure of the network by constructing a network structure. The method aims to dynamically reveal changes in network topology and spatial organisation to elucidate the coupling process and evolutionary mechanism of functional evolution [37]. This study conducts a patent intelligence analysis with the help of social network analysis methods and uses UCINET 6.0 software to process the co-occurrence matrices and draw visual maps for the centrality, core-edge and clustering analyses of patent information. Given the intricate network relationships among patent applicants, conventional multiple regression analyses cannot effectively explore nonindependent relationship data, the use of ordinary least squares (OLS) regression makes it difficult to effectively explore non-independent relational data and may lead to biased estimates [38].Quadratic assignment procedure (QAP) analysis, as a nonparametric permutation test for relational data, can be used to explain patterns between relationships [39], achieve hypothesis testing of “relationship–relationship” relationships, Also, the QAP performs randomised non-parametric tests for similarity/correlation of two or more matrices to avoid problems such as endogeneity and spurious correlation and to produce relatively unbiased statistical results. The method is run by randomising the columns and rows of the dependent variable matrices and performing the regression process many times (usually two thousand times) to estimate the coefficients and p-values to present the relationship between the dependent and independent variable matrices.As a more established measure, it is used in this study to examine the impact of multidimensional proximity factors on the collaborative innovation performance of different cities or different industries in a given region [40,41].In addition, to verify the robustness of QAP analysis results, the exponential random graph model in the Statnet package of R software was used to test the research results.

### 3.3 Variable measurement

#### 3.3.1 Dependent variables.

Drawing on the methods of Zhang et al. [42] and Su et al. [43] in terms of the method of dependent variable measurement, this study takes cooperation and innovation performance as the objects of research and takes the number of jointly filed patents in the sports industry between regions as the measurement standard. We construct the interregion cooperation matrix *CopN*_*ij*_, which reflects the number of jointly applied patents between region *i* and region *j*. Further, we have standardised the data for the inter-regional cooperation matrix *CopN*_*ij*_ by deviation standardisation, highlighting the relative differences in the data. This is due to the fact that for the QAP method, the comparison focuses on the differences in the attribute values of the different options relative to the mean or extreme values rather than the absolute values. Therefore, by standardising the deviations, the relative differences between the options can be better reflected for analysis and decision making. The standardisation of deviations helps to reduce the interference of extreme values (outliers) in the analysis results, making the results more robust. The calculation formula is as follows:


$$Pat_{ij}=\;\frac{CopN_{ij}\;-\;Min(CopN_{ij})}{Max(CopN_{ij})-Min(CopN_{ij})}$$ (1)

where *Pat*_*ij*_ denotes the measure of the dependent variable with a range of 0–1; and *Max*(*CopN*_*ij*_) and *Min*(*Cop*N_ij_) refer to the maximum and minimum values of the interregional cooperation matrix, respectively.

#### 3.3.2 Independent variables.

Boschma classified proximity into geographic, organisational, cognitive and institutional proximity [12], and this classification is widely accepted. Research on organisational proximity is usually used to study interorganisational coinnovation networks such as enterprises, universities, and research institutions. However, this paper focuses on nationwide intercity coinnovation networks, so the influence of organisational proximity is excluded. On the basis of Boschma’s findings, cities in economic proximity have similar modes of operation in the sports industry capital market, and the cost of capital and preferences tend to converge, prompting the flow of innovation capital between cities in economic proximity. The demand for and supply of innovative talent in terms of the structure and level are closer, thus providing realistic feasibility for the flow of innovative talent. Cities with technological proximity are often more likely to experience industrial agglomeration effects and industrial chain advantages, with frequent innovation activities and active innovation markets, which help promote the rational flow and effective allocation of innovation resources and factors in related fields and industries [44]. Therefore, this study explores the influence effect on the innovation network of the sports industry from five aspects: geography, cognition, system, economy and technology.

(1)Geographic proximity, which is one of the earliest and most frequently studied proximity dimensions, represents the spatial distance between nodes in a cooperative network and reflects the degree of proximity between subjects. Referring to the method of Ruan et al. [44], geographic proximity is calculated via the Earth’s spherical distance formula, which is based on the latitude and longitude and measures the spatial straight-line distance the capital city and the innovative city in thousands of kilometres, as shown in Equation (2). In addition, to reduce the impact of data anisotropy while more accurately measuring the relative changes in the data, the intercity distance is logarithmically processed, with larger values indicating lower geographic proximity.


$$Geo_{ij}=\;=\;R\times \text{ arccos }[\text{sin}\left(lat_{i}\right)\text{sin}\left(lat_{j}\right)+\text{ cos}\left(lat_{i}\right)\text{cos}\left(lat_{j}\right)\text{cos}\left(\left|long_{i}-long_{j}\right|\right)]$$ (2)

where *Geo*_*ij*_ represents the geographical distance between two cities; *long*_*i*_ and *lat*_*i*_ represent the longitude and latitude of city *i*, respectively; *long*_*j*_ and *lat*_*j*_ represent the longitude and latitude of city *j*, respectively; and *R* represents the radius of the Earth, which is 6371 km.

(2)Cognitive proximity, which was originally proposed by Nooteboom [45], is defined as the similarity of the ways in which subjects perceive, account for, understand, and evaluate the world and is described by Boschma [46] as “the possibility that people with the same knowledge base and expertise can learn from each other”. It reflects the extent to which the knowledge base is shared among STI subjects and is regarded as a prerequisite for knowledge cooperation and technology exchange [47]. Cognitive proximity helps regional innovation subjects integrate complementary knowledge and improve the efficiency of knowledge spillover through sharing knowledge, thus promoting diverse and dynamic regional STI networks. In terms of measurement, scholars such as Wang [8] expressed cognitive proximity using the logarithm of the absolute value of the difference in R&D investment funds between two cities, and a larger value indicates lower cognitive proximity.


$$Cog_{ijt}=Ln\left|Exp_{i}-Exp_{j}\right|\;\left(i\neq j\right)$$ (3)

where *Cog*_*ijt*_ denotes the cognitive proximity between city *i* and city *j* in period *t*; *Exp*_*i*_ and *Exp*_*j*_ denote the average amount of science and technology expenditures of city *i* and city *j* in period *t,* respectively; and a smaller difference indicates greater cognitive proximity.

(3)Institutional proximity is another expression of institutional distance, and its definition is derived from Scott’s [48] three-pillar theory of institutions, i.e., the institutional environment consists of a regulatory pillar, a normative pillar, and a cognitive pillar [49]; it builds on Scott’s view by defining institutional distance as the degree of regulatory, normative, and cognitive differences between countries. Among enterprises, universities and research institutions, institutional proximity plays the role of “bonding” various organisations, which promotes more exchanges and mutual learning among innovation actors and results in more stable relationships [50]. Given that there are different administrative levels between cities, a dummy variable approach is used to quantify cities with different administrative levels. In accordance with the methods of Su et al. [51], we assign the capital city a value of 4, the municipality directly under the central government a value of 3, the capital city of the province a value of 2, and the prefecture-level city a value of 1. The institutional proximity matrix is constructed by adding up the assigned values between each two cities and excluding the samples belonging to the same city to obtain the institutional proximity matrix between two cities.


$$Ran_{ij}=Ln\left|Reg_{i}+Reg_{j}\right|\left(i\neq j\right)$$ (4)

where *Ran*_*ij*_ denotes the administrative hierarchical proximity between city *i* and city *j* in period *t*. *Ran*_*i*_ and *Ran*_*j*_ denote the administrative hierarchical assignments of city *i* and city *j,* respectively, in period *t*. A larger value indicates that large and medium–sized cities cooperate more closely, and a smaller value indicates that small and medium-sized cities cooperate more closely.

(4) Economic proximity refers to the proximity of regions in terms of economic scale and economic structure [8]. Science, technology and innovation networks are the result of knowledge search, technological cooperation and flow, and regions with economic proximity usually have similar technological systems, human capital, technological demands and technological absorptive capacity, which is conducive to knowledge exchange and technological cooperation, as well as to the flow of innovative resources and technological spillovers [52]. Drawing on the research of Hu [53], economic proximity is measured via the logarithm of the absolute value of the difference in per capita GDP across cities; the more similar the economic levels of the two places are, the greater the economic proximity is.


$$Eco_{ijt}=Ln\left|Pgdp_{i}-Pgdp_{j}\right|\left(i\neq j\right)$$ (5)

where *Eco*_*ijt*_ denotes the economic proximity between city *i* and city *j* in period *t*, and *Pgdp*_*i*_ and *Pgdp*_*j*_ denote the average GDP per capita of city *i* and city *j,* respectively, in period *t*. The smaller the difference is, the greater the economic proximity.

(5) Technological proximity is a concept first proposed by Jaffe [54] to indicate the degree of similarity in technological dimensions between two subjects. Technological proximity promotes interorganisational knowledge flow, and a similar technological structure facilitates mutual understanding, increased absorptive capacity, and more effective exchange and acquisition of external knowledge [55]. It describes the degree of association and overlap between the core enterprise and related subjects in terms of the cognitive base and technological structure and has an important positive effect on shortening the distance between different firms in the cooperative network and improving cooperative innovation performance [56]. According to previous research, technical proximity is expressed as the logarithm of the absolute value of the difference in the number of patents granted between two cities [8], and a larger value indicates lower technological proximity.


$$Tec_{ijt}=Ln\left|Pat_{i}-Pat_{j}\right|\left(i\neq j\right)$$ (6)

where *Tec*_*ijt*_ denotes the technological proximity between city *i* and city *j* in period *t*, and *Pat*_*i*_ and *Pat*_*j*_ denote the average number of patents granted to city *i* and city *j*, respectively, in period *t*. The smaller the difference is, the greater the technological proximity.

#### 3.3.3 Control variables.

In addition, this study introduces two control variables. Previous research has shown that administrative boundaries and administrative hierarchies have a significant effect on the evolution and formation of innovation cooperation networks in the context of China’s specific national conditions [57]. To measure this effect, two proxy variables are introduced: first, “administrative boundary”, which is set to 1 if the two cities are located in the same province, and 0 if they are located in different provinces; and second, “administrative level”, which is set to 1 if at least one of the two cities is a provincial capital city, and 0 if the two cities are located in different provinces. If at least one of the two cities is a provincial capital city, it is set to 1; if the two cities do not contain provincial capital cities, it is set to 0.

The influencing factor model of China’s sports industry innovation network is constructed as follows


$$Pat_{ij}=\left(Geography_{ij};Cognition_{ij};Institution_{ij};Economy_{ij};Technology_{ij};Border_{ij};Grade_{ij}\right)$$ (7)

Both sides of Eq. (7) are presented as relational data in the form of a matrix, and

the specific measures of each variable are shown in Table 1.

**Table 1:** Variable selection and measurement.

Variable type	Variable name	Variable symbol	Measurement method
Outcome variable	Collaborative innovation performance	*Pat*_*ij*_	Number of joint patent applications in the sports industry category between cities (Min–Max Normalisation)
Conditional variables	Geographic proximity	*Geography*_*ij*_	Logarithm of the geographic straight-line distance between cities
	Cognitive proximity	*Cognition*_*ij*_	Logarithm of the absolute value of the difference in R&D input expenditures between cities
	Systemic proximity	*Institution*_*ij*_	Quantification of cities with different administrative levels using dummy variables
	Economic proximity	*Economy*_*ij*_	Logarithm of the absolute value of the difference in per capita GDP between cities
	Technological proximity	*Technology*_*ij*_	Logarithm of the absolute value of the difference in the number of patents granted between cities
Control variables	Administrative boundary	*Border*_*ij*_	Whether the partner city is located in the same province
	Administrative level	*Grade*_*ij*_	Whether the partner city contains provincial capitals

## 4. Results

### 4.1 Analysis of the Cooperative Innovation Network

**Fig 1:**
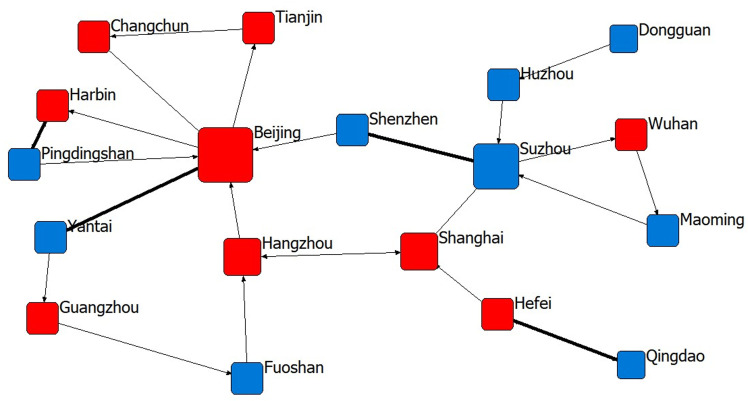
The sports industry innovation network of 11th Five-Year Plan period.

Given that relevant studies at home and abroad usually use a window period of 3–5 years to study cooperative innovation activities [58], to accurately reflect the evolution of the cooperative innovation network of the new energy industry from the perspective of multidimensional proximity, this study adopts the 5-year period of the national development plan to divide the observation time into four stages. Therefore, the joint patent data of different applicant subjects in cities in these four phases were collected, and these data were organised and summarised. Subsequently, we used UCINET 6.0 software to map the collaborative innovation network at each stage (Fig 1),with the thickness of the connecting lines indicating the number of collaborations, and node sizes presented in degree-centred degrees, and descriptive analyses of the changes in the parameters of each stage and the relevant features of the network were also performed.

**Fig 2:**
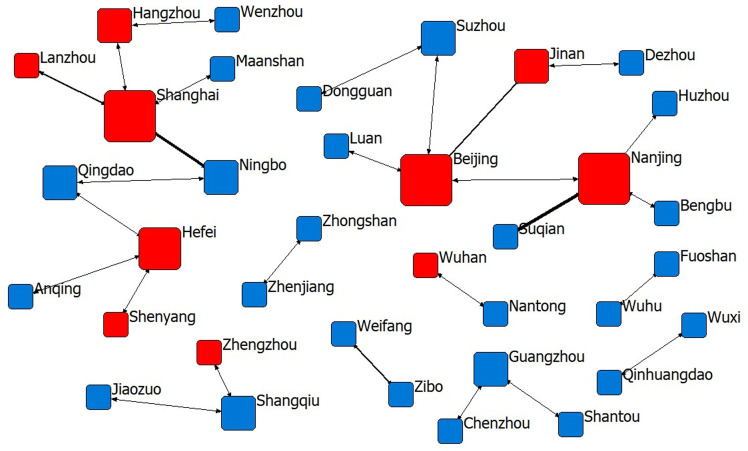
The sports industry innovation network of 12th Five-Year Plan period.

**Fig 3:**
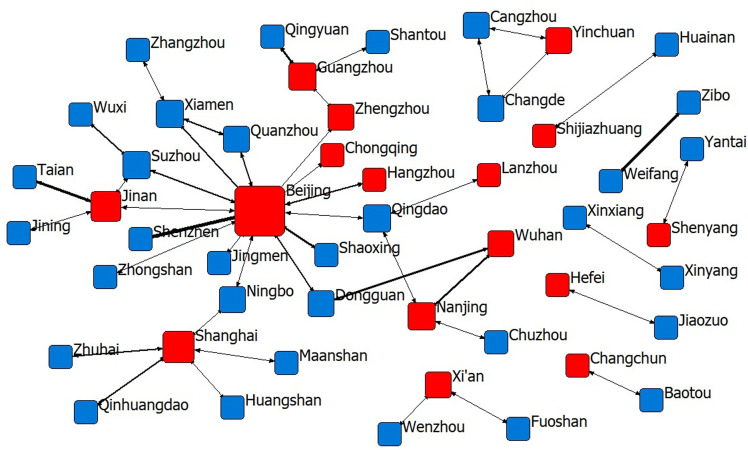
The sports industry innovation network of 13th Five-Year Plan period.

**Fig 4:**
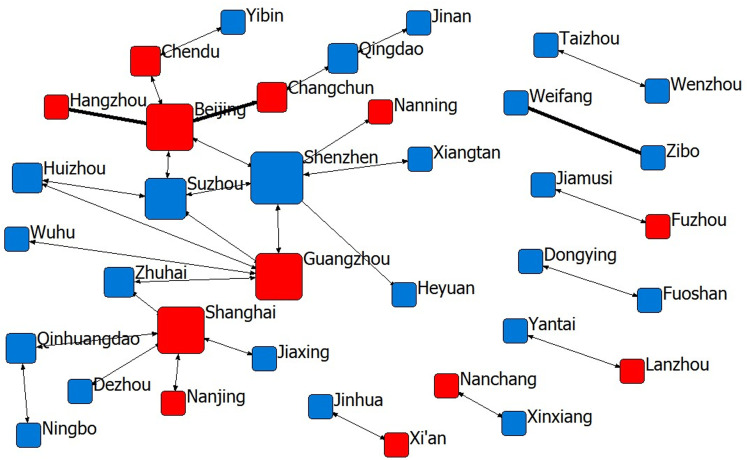
The sports industry innovation network of 14th Five-Year Plan period.

From Figs 1–4 and Table 2, it can be seen that among the sports industry invention patent networks authorised during the “11th Five-Year Plan” period, although the number of cooperating cities, the number of patents disclosed and the number of links are the lowest among the four phases of the Five-Year Plan period, the density of the network, the average length of the paths and the cohesion index are all the greatest; that is, the innovation links of the sports industry in the 18 cities are the tightest and the most cohesive. Among them, Beijing and Suzhou have the highest degree of centrality, indicating that these two cities have greater importance in the innovation network. From the “12th Five-Year Plan” period onwards, due to the gradual increase in the demand for innovation in the sports industry, an increasing number of cities begin cooperating in innovation, and the network density, average path length and cohesion index decrease, among which Beijing, Shanghai and Nanjing have the highest importance in the innovation network during the “12th Five-Year Plan period” and the situation of “multi-core development” has been presented. With the promulgation of “Several Opinions of the State Council on Accelerating the Development of Sports Industry and Promoting Sports Consumption” in 2014, the development of China’s sports industry has entered a period of rapid development, and the public’s enthusiasm for sports consumption has been further enhanced, and more and more attention has been paid to the innovation content of sports and fitness products. In the “13th Five-Year Plan” period, cooperation and innovation among industries, universities and research institutes have gradually become an important way for sports enterprises to improve the technological content of their products. As the political, economic and cultural centre of China, Beijing has played a spillover effect of technological innovation during this period, and has an important core position among the 49 innovative cities in the “13th Five-Year Plan” period, which also makes the degree of network centrality in the “13th Five-Year Plan” period the highest among the four Five-Year Plans.

**Table 2:** Statistics of basic parameters of cooperative innovation networks at each stage.

Indicators	“11th Five-Year Plan” period	“12th Five-Year Plan” period	“13th Five-Year Plan” period	“14th Five-Year Plan” period
Public time interval (year)	2006–2010	2011–2015	2016–2020	2021–2023.12
Number of cooperating cities (number)	18	37	49	38
Number of jointly disclosed patents (units)	32	76	158	68
Network density	0.095	0.053	0.060	0.054
Network centricity	0.169	0.038	0.250	0.077
Number of links	25	54	88	62
Average path length	4.178	2.515	3.126	3.605
Cohesion index	0.271	0.081	0.161	0.147

As of December 2023, in the innovation cooperation network in the “14th Five-Year Plan “ period, the cities Beijing, Guangzhou, Shenzhen and Shanghai all have a high degree of centrality, indicating that the structure of the main body of innovation cooperation in the sports industry is gradually becoming more dispersed, the core cities of the main body of cooperation is concentrating in the four super first-tier cities in China, the complexity of the cooperation relationship is gradually increasing, and the development situation of technology spillover is gradually forming on the basis of the economic strength of these cities. In addition, as shown by the red nodes in Figs 1–4, the provincial capitals and municipalities are moving closer to the centre of the network, and the cooperation links with neighbouring cities are becoming increasingly closer, indicating that these cities are rich in economic, scientific, technological, talent and other innovation resources and will achieve increasingly important control and dominance in the innovation network of China’s sports industry.

### 4.2 QAP multiple regression analysis

The model of this study includes five explanatory variables, two control variables and one explanatory variable, and all these variables are in matrix form. In accordance with the variable measurement method described in the previous section, we calculated and organised the data collected for each type of variable accordingly. The descriptive statistics of the variables in the matrix are shown in Table 3. Using UCINET 6.0 software, we carried out a QAP multiple regression analysis on the collated multidimensional proximity data to investigate the degree of influence of the multidimensional proximity factors on the cooperation and innovation performance of the sports industry among cities in each stage from the “11th Five-Year Plan” period to the “14th Five-Year Plan” period. The results of the QAP regression analysis are shown in Table 4.

**Table 3:** Descriptive statistics of variables.

Variable	Mean	SD	Min	Max	*N*	Mean	SD	Min	Max	*N*
	“11th Five-Year Plan” period					“12th Five-Year Plan” period				
*Pat*_*ij*_	0.064	0.239	0	1	306	0.071	0.251	0	1	1332
*Geography*_*ij*_	6.782	0.380	0	7.613	306	6.801	0.391	0	7.627	1332
*Cognition*_*ij*_	10.435	3.062	0	11.961	306	10.094	2.895	0	12.131	1332
*Institution*_*ij*_	1.227	0.361	0.693	2.079	306	0.993	0.519	0.693	2.079	1332
*Economy*_*ij*_	10.558	2.473	0	11.790	306	11.280	2.479	0	13.539	1332
*Technology*_*ij*_	9.092	2.608	0	11.564	306	9.955	2.725	0	11.580	1332
*Border*_*ij*_	0.078	0.248	0	1	306	0.096	0.291	0	1	1332
*Grade*_*ij*_	0.601	0.449	0	1	306	0.595	0.493	0	1	1332
	“13th Five-Year Plan” period					“14th Five-Year Plan “period				
*Pat*_*ij*_	0.078	0.259	0	1	2353	0.076	0.249	0	1	1406
*Geography*_*ij*_	6.823	0.376	0	7.691	2353	6.819	0.389	0	7.668	1406
*Cognition*_*ij*_	10.233	2.992	0	12.288	2353	10.209	2.926	0	12.231	1406
*Institution*_*ij*_	1.011	0.477	0.693	2.079	2353	1.041	0.491	0.693	2.079	1406
*Economy*_*ij*_	11.133	2.475	0	13.529	2353	11.185	2.602	0	13.535	1406
*Technology*_*ij*_	9.906	2.760	0	11.601	2353	9.882	2.747	0	11.592	1406
*Border*_*ij*_	0.065	0.249	0	1	2353	0.078	0.261	0	1	1406
*Grade*_*ij*_	0.488	0.498	0	1	2353	0.535	0.465	0	1	1406

**Table 4:** Results of QAP regression analysis.

Variable	“11th Five-Year Plan” period		“12th Five-Year Plan” period		“13th Five-Year Plan” period		“14th Five-Year Plan” period	
Unstand-coef	Stand-coef	Unstand-coef	Stand-coef	Unstand-coef	Stand-coef	Unstand-coef	Stand-coef
Geographic proximity	‒0.133^*^	‒0.273^*^	‒0.056^**^	‒0.279^**^	0.215	0.337	0.037	0.230
Cognitive proximity	0.000	0.022	0.000^**^	0.185^**^	0.001^**^	0.257^**^	0.000^**^	0.095^**^
Systemic proximity	‒0.019^*^	‒0.056^*^	0.025^***^	0.082^***^	0.049^***^	0.123^***^	0.053^***^	0.204^***^
Economic proximity	‒0.001^***^	‒0.101^***^	‒0.000^**^	‒0.002^**^	‒0.001^**^	‒0.033^**^	‒0.000^*^	‒0.192^*^
Technological proximity	0.006	0.123	‒0.000	‒0.141	0.000	0.054	‒0.000^**^	‒0.029^**^
Administrative boundary	0.311^*^	0.186^*^	0.043	0.073	0.403	0.278	0.201	0.278
Administrative level	0.189	0.196	0.003	0.006	0.017	0.023	‒0.087^**^	‒0.173^**^
*R* ^2^	0.315		0.328		0.365		0.332	
*N*	306		1332		2352		1406	

Note:

* *P* < 0.1,

** *P* < 0.05,

*** *P* < 0.01;“Stand-coef” means Standardised coefficient;“Unstand-coef” means Unstandardised coefficient

According to the results of the QAP multiple regression analysis in Table 4, in the “11th Five-Year Plan “ period (2006–2010), economic proximity is the most significant, followed by administrative hierarchy, institutional proximity, administrative boundaries and geographic proximity, of which institutional proximity and economic proximity have a significant inhibitory effect on the cooperation among sports industry patents. Institutional proximity and economic proximity have significant inhibitory effects on sports industry patent cooperation. During the “11 Five-Year Plan” period, innovation cooperation among cities in China favoured small and medium-sized cities with similar levels of economic development, the cooperation efficiency among large cities was not as good as that among small and medium-sized cities, and the cooperation efficiency among cities in the same province and in similar geographical locations was greater. In addition, technological proximity is not significant, indicating that there may be technological competition, knowledge reserve competition, product market competition, etc., between enterprises and universities in the region, which leads to an increasing number of enterprises choosing to perform R&D on their own or in cooperation with their parent companies and subsidiaries. Cognitive proximity is not significant, indicating that the sharing of the knowledge base among S&T innovation actors is low and that effective knowledge spillover has not yet occurred.

During the “12th Five-Year Plan” period (2011–2015), geographic proximity continues to have a positive effect on innovation in the sports industry, but unlike the “11th Five-Year Plan “ period, institutional proximity has shifted from inhibition to promotion, indicating that as human and technological resources for innovation gather in first- and second-tier cities, innovation cooperation among large and medium–sized cities has become closer. Unlike the “11th Five-Year Plan” period, institutional proximity has shifted from inhibition to promotion, indicating that innovation cooperation in large and medium–sized cities has become increasingly close as human and technological resources for innovation have gathered in first- and second-tier cities. In addition, the facilitating effect of cognitive proximity suggests that the cognitive gap significantly promotes interregional technological cooperation, as China’s consumer groups further increase their requirements for the technological content of products such as sporting goods and sports services. A large cognitive gap is conducive to the exchange of heterogeneous knowledge among innovative subjects, as well as the generation of new knowledge and new technologies, thus promoting innovation cooperation and technology exchange between regions. Moreover, cities with similar economic strengths are more likely to contribute to the output of innovative technologies. The main cities cooperating are no longer limited to cities in the same province but have shifted to cities in provinces that are closer geographically. At the same time, innovation cooperation among cities is more inclined towards cities with large differences in economic development levels.

During the “13th Five-Year Plan “ period (2016–2020), both geographical proximity and administrative boundaries, which were significant in the early periods, do not play a significant role in promoting cooperation and innovation in the sports industry, indicating that geographical location is no longer a consideration for the cooperation of relevant innovation entities. Instead, the importance of cognitive proximity, institutional proximity and economic proximity increases, i.e., the widening of the cognitive gap further facilitates innovation cooperation between cities, whereas the concentration of innovation resources in first-tier cities increases further. In addition, there are no significant differences in technology or administrative hierarchies. In the first half of the “14th Five-Year Plan” period (2021-2023), the importance of cognitive proximity slightly decreases, whereas the importance of institutional proximity and economic proximity further increases. Moreover, technological proximity and the administrative hierarchy create disincentives for intercity innovation, i.e., cities with similar technological capabilities become more prone to innovation cooperation, whereas cooperation between small and medium-sized cities gradually increases.

### 4.3 Robustness test

To verify the robustness of QAP analysis results, the exponential random graph model in the Statnet package of R software was used to test the research results.The model is based on a network relationship, which is a more important relational data measurement model at present [59], and the results are shown in Tab. 5. A comparison of the results in Table 4 and Table 5 reveals that the significance of the impact of the different types of proximity as well as the related control variables on the performance of cooperation and innovation in China’s sports industry basically remains the same. As a result, it can be concluded that the study results are robust and effectively verifies that multidimensional proximity has important value in the sports industry’s industry-university-research cooperation and innovation in different cities.

**Table 5:** Robustness test.

Variable	“11th Five-Year Plan” period	“12th Five-Year Plan” period	“13th Five-Year Plan” period	“14th Five-Year Plan” period
Geographic proximity	‒ *	‒^**^	+	+
Cognitive proximity	+	+^***^	+^**^	+^**^
Systemic proximity	‒^ * ^	+^***^	+^**^	+^***^
Economic proximity	‒^***^	‒^***^	‒^**^	‒^ * ^
Technological proximity	+	+	+	‒^**^
Administrative boundary	+^**^	+	+	+
Administrative level	+	+	+	‒**

## 5. Discussion

To date, the research on innovation networks has gradually shifted from conceptual research to a more precise quantitative analysis stage. The current research is mainly qualitative normative research and empirical research, and scholars have conducted extensive research on innovation networks in different countries and industry types. This paper explores the impact of multidimensional proximity on the development and evolution of cooperative innovation networks in the sports industry in China from the perspective of proximity. Owing to the continuous development and evolution of cooperative innovation networks in the sports industry, the development direction and goals of the nodes within the network are also changing, resulting in corresponding changes in the impact of multidimensional types of proximity on the cooperative innovation network at different stages of development. According to the above QAP multiple regression results, combined with the status quo of China’s sports industry and previous research results, the following analysis results are obtained:

(1) Geographical proximity. During the periods of the “11th Five-Year Plan “ and the “12th Five-Year Plan”, i.e., 2006–2015, the cooperation and innovation of China’s sports industry were affected by geographical proximity. The period was more inclined towards similar geographical areas within the province, while the “12th Five-Year Plan” period gradually showed spreading to the neighbouring provinces and regions but was still generally concentrated in a local area, which is consistent with the conclusion of the study by Wang et al. [5]; that is, the distribution of patents in China’s sports industry varied greatly before 2017, and the status quo of the imbalance in the innovation capacity of the sports industry was more prominent. However, with the introduction of the “No. 46” document in approximately 2017 and the subsequent relevant sports industry support policies, coupled with the rapid development of China’s transport network and communication technology, innovation cooperation in the sports industry gradually began to break geographical limits, and the provinces with strong innovation resources gradually began to drive improvements in the innovation capacity of geographically distant provinces. Research has shown that since 2018, the high agglomeration of the innovation capacity of the sports industry in different provinces of China has become increasingly obvious, and the representative provinces are Jiangsu, Hunan, Hubei, Anhui, Shandong, etc.[5], which also indicates that, through the spillover of innovation technology in the sports industry, innovation technology and capacity have gradually become more balanced geographically. In the next step of the spatiotemporal evolution of the innovation network, the innovation of the sports industry agglomeration should be accelerated, and an efficient technology information-sharing platform should be built on the basis of regional real economy industry agglomeration and enhance the efficiency of inter-regional sports industry innovation cooperation.
(2) Cognitive proximity. Except for the “11th Five-Year Plan” period, there was a significant positive effect from the “12th Five-Year Plan” period to the “14th Five-Year Plan” period, showing an inverted U-shaped relationship. This shows that from the 12th Five-Year Plan period onwards, the degree of knowledge base sharing of sports science and technology innovation subjects in different cities in China deepened, the gap in cognitive ability intensified the cooperation efficiency among different innovation subjects, and the sharing of related technologies improved knowledge spillover efficiency. Knowledge information is the source of inspiration for technological innovation, especially for the sporting goods manufacturing technology industry, which has a high technological threshold, and not everyone has the cognitive basis for technological innovation cooperation; similar cognitive proximity decreases the communication and cooperation distance, but in the sports industry, differences in cognitive proximity promote communication and cooperation between innovative subjects. At present, China’s sporting goods network shows irrational distribution of innovation resources in the manufacturing industry, stadium construction industry and sports service industry and there are still technical barriers in key areas, which is also consistent with the findings of Wang et al. [60]and He et al. [61]. The inverted “U”-shaped impact trend suggests that this problem is gradually improving and that the technology market is moving in a more benign direction.
(3) System proximity: As shown in Fig 2, during the “11th Five-Year Plan” period, China’s sports industry innovation cooperation was mainly concentrated in small and medium-sized cities, with the continuous influx of technology, talent, capital, information and other essential resources for innovation to large cities, a “siphon effect” has been formed. This comes from the resource agglomeration effect and scale economy of large and medium-sized cities, the pull of market demand, policy and platform advantages, large events and brand effect, information technology and data advantages, as well as the preference and flow of talents, prompting the gradual deepening of innovation cooperation in sports industry among large and medium-sized cities. Take the cooperation between provincial capitals as an example, during the period from the “12th Five-Year Plan” to the “14th Five-Year Plan”, the innovation connection between different provinces has become closer and closer, and the phenomenon of cooperation between provincial capitals of the eastern region and those of the central and western regions has also gradually increased.However, it is important to note that too much institutional proximity can also lead to the negative effects of “institutional inertia”, which may have a negative impact on new knowledge learning and innovation.Therefore, through the introduction of dynamic incentive policies for the sports industry, flexible innovation management processes and flexible property rights allocation mechanisms, we can promote cross-institutional knowledge flows and improve the existing benefit distribution mechanism, so that all parties in the co-operation can flexibly adjust the distribution rules according to different institutional bases, thus stimulating the co-operation and innovation motivation of the participants of the sports industry’s innovation activities in cities with different institutional environments.
(4) Economic proximity: During the “11th Five-Year Plan” period, the wider the gap in economic strength between cooperating cities, the greater the inhibition of innovation output; i.e., innovation cooperation was concentrated among cities with comparable economic strength, but since then, economic strength ceased to be a factor for intercity cooperation and innovation. Economic proximity promotes close industrial links and frequent economic interactions between different cities in the collaborative development zone within the province, which meets the real needs of different cities for cooperation and innovation. Owing to the differences in industrial relevance and resource allocation between neighbouring cities, there is room for cooperation in technology research and development and innovation among innovation agents. Regions with economic proximity usually have similar technological systems, human resources, technological needs and absorptive capacity, which are conducive to knowledge exchange and technological cooperation. Research by scholars such as Scherngell et al. [62]and Ren et al. [63] also confirmed this view that the lower the economic proximity between regions, i.e., the greater the difference in economic scale and structure, the less conducive it is for the backwards regions to absorb and introduce advanced knowledge and technology, and the lower the possibility of their scientific research cooperation and technology exchange.Based on this, the establishment of a cross-regional joint R&D platform, the setting up of a technical consultant mechanism, the implementation of the “technology for market” model, the development of tourism sports, ecological sports, rural sports and other special sports economy, and the creation of attractive regional sports brands and events are powerful ways to promote the technological innovation and exchange of the sports industry among cities with different economic strengths. These are powerful ways to promote technological innovation and exchange in sports industry among cities with different economic strengths.
(5) Technology proximity: Between the 11th and 13th Five-Year Plan periods, innovation technology gaps did not have a significant effect on innovation cooperation outputs, whereas in the 14th Five-Year Plan period, cities with similar innovation technology capabilities cooperated more closely with each other, which significantly increased cooperation and innovation performance. In the technology cooperation and innovation network of the sports industry, innovation subjects should join hands to overcome technical difficulties, develop new technologies with independent intellectual property rights and breakdown technical barriers.

However, judging from the current development situation, due to the “chasm effect” brought about by the gap between the technological strength of different cities, the inequality between innovation ability and demand, the lack of balanced benefit sharing mechanism, the gap between innovation talents and management ability, the uneven support of regional policies and other realities, China’s sports industry innovation and cooperation, represented by cities, has only gradually realised the cooperation and aggregation with similar innovation and technological strength since 2021. The effective innovation cooperation between cities with too big difference in technological strength still faces serious challenges due to the limitation of technological divide and other factors. In the future, through building joint innovation platforms, setting up special funds for regional co-development, strengthening industrial chain linkage and collaboration, establishing technology export and sharing mechanisms, improving benefit sharing and protection mechanisms, improving the technology undertaking capacity of technologically disadvantaged cities, and developing the “enclave economy” mode of technologically strong +  weak cities, it is the best way to narrow the gap in technological capacity of urban sports industry and promote the development of large-scale sports industry. It is an effective way to narrow the gap between the technological capabilities of cities in the sports industry and promote the performance of cooperation and innovation in the sports industry in large, medium and small cities.

## 6. Conclusion

### 6.1 Conclusions

On the basis of the multidimensional proximity perspective, this study divides 2006-2023 into four research phases, with each five-year plan period as a phase, and by observing the changes in these four phases, we can depict the development and evolution of China’s cooperation and innovation network in the sports industry. This study relies on UCINET 6.0 software to visualise the cooperation and innovation network and performs a QAP multiple regression analysis on the data. We explored the factors driving the evolution of interregional sports industry cooperation and innovation networks from the perspectives of network structure and multidimensional proximity. On the basis of the research results, we draw the following conclusions:(1)Research on the characteristics of cooperation and innovation networks in the sports industry revealed that through rapid development from the “11th Five-Year Plan” period to the “14th Five-Year Plan” period, the scale of China’s sports industry cooperation and innovation network has expanded, the interregional links have become increasingly close, the number of interregional joint patent applications has grown rapidly, and a grid-based cooperation situation with multiple innovation bodies, such as Beijing, Shanghai, Guangzhou and Shenzhen, has gradually formed in place of the “one-place-led” style in the early days. Moreover, the number of members in this cooperation and innovation network is increasing, the interregional links are becoming increasingly close, and after the “12th Five-Year Plan” period, the cohesion of the network has increased, along with the complexity of the interregional cooperation relationship.(2)Through empirical research, it was found that from 2006 to 2015, innovation cooperation in the sports industry was more likely to occur between geographically neighbouring regions, but since the “13th Five-Year Plan” period, innovation cooperation in the sports industry has gradually broken geographic limits. Since 2011, the knowledge base sharing of sports science and technology innovation subjects in all provinces and regions in China has further deepened, the gap in cognitive ability has exacerbated the efficiency of cooperation between different innovation subjects, and the cooperation between large and medium–sized cities has gradually deepened. In addition, the wider the gap in economic power between the cooperating cities is, the greater the dampening effect on innovation output, i.e., innovation cooperation is concentrated among cities with comparable economic power. In the “14th Five-Year Plan” period, however, closer cooperation between cities with similar innovative and technological capabilities can significantly increase the innovation outputs of cooperation.

### 6.2 Countermeasure Suggestions

On the basis of the above research conclusions and the reality of the development of the sports industry innovation network in China, the following countermeasures are proposed:

(1) Build a regional sports industry cooperation platform and promote the development of sports industry strategic alliances. Although the industrial cluster effect formed by national and provincial sports industry bases in recent years has promoted the development of sports industry cooperation and innovation networks, the importance of geographical proximity has gradually weakened with the rapid development of China’s transportation and communication technologies and the constant impact of emerging technologies such as blockchain, artificial intelligence and big data. On the other hand, cognitive proximity has always been significant in promoting the development of the sports industry, which indicates that the distribution of innovation resources in China’s sporting goods manufacturing industry, stadium construction industry and sports service industry is still urgently needed. Therefore, the government’s macrocontrol and the market’s regulation should be brought into play to strengthen the awareness of knowledge sharing and innovation cooperation among regions, promote the free flow of scientific and technological innovation resources, and replace regional competition with regional cooperation.
(2) With regard to institutional proximity, the focus is on giving play to the driving role of strong resource cities and leading innovation cooperation among small and medium-sized cities. The complementarity of the advantages between cities with different technological innovation resources should be strengthened, and an innovation alliance between small and medium-sized cities should be created. Moreover, according to local conditions, a differentiated sports industry structure in line with regional advantages should be established, the optimisation and upgrading of the sports industry structure in the region should be accelerated, local protectionism should be broken, a correct sense of competition should be established, and the exchange of technology, talent and resources between regions should be strengthened. In terms of economic proximity, innovation cooperation between cities with similar economic strength should be promoted, which can highlight the resource allocation and competitive advantages of different cities, promote synergistic cooperation and heterogeneous and complementary relationships in the sports industry chain, support the upgrading of the sports industry and high-quality development of the region, and provide a suitable economic foundation and social environment for the regional collaborative innovation system. In terms of technological proximity, the regional technological innovation cooperation mechanism between regions should be further improved to promote the exchange and market transactions of technological innovation factors and resources. Cross-city industry-academia-research institutes are encouraged to carry out horizontal and vertical cooperation to increase the overall strength of regional technological innovation. Moreover, further emphasis should be placed on strengthening the construction of regional collaborative innovation platforms between cities with similar technological capabilities to promote the integration of innovation resources and factors through policy guidance and platform docking, thereby enhancing innovation efficiency. In terms of cooperation and innovation behaviours, the establishment of a new type of collaborative innovation subject relationship will promote the construction of regional collaborative innovation networks in different provinces and regions to increase the activity and effectiveness of innovation cooperation and enhance the regional collaborative innovation performance of the sports industry.

## 7. Limitations and prospects

This study takes Chinese sports industry invention patents as the data sample, constructs a topological map of urban sports industry cooperation and innovation networks from the “11th Five-Year Plan” period to the “14th Five-Year Plan” period, and analyses the internal structure pattern of the network. Moreover, QAP multiple regression analysis is used to investigate the influence of geographical, cognitive, institutional, economic and technological proximity on the performance of sports industry cooperation and innovation at different stages. However, the following research limitations remain: (1) Due to the lag in the development of sports industry statistics in Chinese cities, collaborative innovation performance can be measured only in the form of patents, which may not comprehensively reflect the innovation situation of the sports industry in China. (2) This paper constructs a cooperative innovation network of the Chinese sports industry in each five-year period, but the time span can be further reduced. (3) This paper constructs a cooperative innovation network only at the city level, whereas from a more microscopic perspective, the cooperative network among innovation subjects in different cities may present different forms. Therefore, in future research, we will make the following breakthroughs: (1) collect relevant data that can more accurately characterise the innovation performance and innovation level of China’s sports industry to make the research results more rigorous; (2) construct a cooperation and innovation network of China’s sports industry in one-year or two-year periods and make longitudinal comparisons under the time dimension to observe the internal change trends in the cooperation and innovation network structure more clearly; and (3) take the main innovation actors in China’s sports industry, such as different enterprises, universities or research institutes, as the investigation objects and construct a cooperative innovation network in different periods to analyse the technological flow and changes in China’s sports industry from a more microscopic point of view.

## Supporting information

S1 Data (ZIP)
